# Quantitative Assessment of Human Health Risks Associated with Heavy Metal and Bacterial Pollution in Groundwater from Mankweng in Limpopo Province, South Africa

**DOI:** 10.3390/ijerph21111489

**Published:** 2024-11-09

**Authors:** Tsolanku Sidney Maliehe, Nelisiwe Mavingo, Tlou Nelson Selepe, Peter Masoko, Frederick Mokibelo Mashao, Neville Nyamutswa

**Affiliations:** 1 Department of Water and Sanitation, University of Limpopo, Private Bag X1106, Polokwane 0727, South Africa; 202047288@keyaka.ul.ac.za (N.M.); tlou.selepe@ul.ac.za (T.N.S.); 2 Department of Biochemistry, Microbiology and Biotechnology, University of Limpopo, Private Bag X1106, Polokwane 0727, South Africa; peter.masoko@ul.ac.za; 3 Department of Geography, University of Limpopo, Private Bag X1106, Polokwane 0727, South Africa; frederick.mashao@ul.ac.za; 4 Center for Global Change, University of Limpopo, Private Bag X1106, Polokwane 0727, South Africa; 5 Capricorn District Municipality, P.O. Box 4100, Polokwane 0727, South Africa; nyamutswan@cdm.org.za

**Keywords:** groundwater, heavy metals, heavy metal pollution index, bacteria, health risk assessment

## Abstract

Heavy metal and microbial pollution in groundwater raises health concerns due to its adverse effects. This study aimed to assess the health risks associated with heavy metal and bacterial pollution in groundwater in Mankweng. Heavy metals and *Escherichia coli* were detected using inductively coupled plasma mass spectrophotometry and a Colilert system, respectively. The heavy metal pollution index (HPI) and non-carcinogenic and carcinogenic risks were computed. The β-Poisson dose–response model was employed to predict the probability of *E. coli* infection. The metals’ concentrations were all within the permissible limits of the South African National Standard (SANS). However, Pb levels at sites E and G exceeded the World Health Organization (WHO) guideline (≤0.01 mg/L). HPI values were all less than 100, indicative of low contamination. The hazard quotient values were all less than 1, except for vanadium. The cumulative cancer risk ranged between 3.06 × 10^−5^ and 1.81 × 10^−4^ and between 3.55 × 10^−5^ and 2.20 × 10^−4^ for adults and children, respectively. *E. coli* was only detected at site L. The annual risk of *E. coli* infection exceeded the WHO risk limit of 10^−4^. The results underscore the need for the regular monitoring of groundwater.

## 1. Introduction

The consumption of high-quality water is strongly associated with improved health outcomes [1]. However, in developing countries such as South Africa, only the water supplied by the municipalities is measured against the national and international standards for drinking water to ensure its fitness for consumption or recreational use [2]. The South African National Standard (SANS) sets compliance limits for groundwater quality to safeguard humans, whereas the World Health Organization (WHO) sets global guidelines [3,4]. Despite these well-known guidelines, there have been scant attempts to assess groundwater quality from boreholes, especially in peri-urban and rural regions [5]. Recently, the most concerning form of groundwater contamination is the occurrence of heavy metals [6], pharmaceuticals [7], and pathogenic microorganisms, such as coliforms and *Escherichia coli* [8].

Heavy metals are regarded as one of the significant pollutants of groundwater, with some metals reported to exceed set standard limits in some places, especially in developing countries [9]. Anthropogenic activities and rock weathering are identified as the dominant sources of heavy metal contamination [10]. Although most of them are essential for various biochemical and physiological functions, heavy metals such as copper (Cu), cobalt (Co), lead (Pb), chromium (Cr), cadmium (Cd), nickel (Ni), arsenic (As), and zinc (Zn), often reported in groundwater in the Limpopo province of South Africa [11,12,13], can pose a significant ecological and human health threat due to their high toxicity, persistence, and non-biodegradable nature [14]. Human exposure to these metals can result in detrimental health complications, as they are teratogenic, carcinogenic, and mutagenic, even at very low concentrations [15]. For example, the consumption of excessive Cd in drinking water has been reported to cause carcinogenic effects, bone damage, and renal infection in humans [16], whereas the consumption of iron-contaminated groundwater has been strongly linked to fatigue and joint inflammation [17].

The heavy metal pollution index (HPI) is one of the mathematical rating tools commonly employed to ascertain the degree and effects of heavy metals in groundwater [18]. This tool simplifies the reporting of sophisticated groundwater quality datasets by summarising them into a single unit that can describe their overall quality [19]. By facilitating the easy interpretation of large water quality datasets, the HPI bridges the gap between laymen and groundwater quality experts [20]. The HPI is based on established water quality guidelines and steps, including heavy metal selection, the manipulation of raw data into a common scale, providing weights to the metals of interest, and lastly, the aggregation of subindex values [21]. Thus, several researchers have successfully utilised the HPI to assess the quality of groundwater [22,23,24].

Recent reports have highlighted the health risks posed by waterborne pathogens, emphasising the importance of microbial risk assessments to ensure safe drinking groundwater [25]. The presence of high-risk pathogens such as coliforms (i.e., *Salmonella* spp. and *Escherichia coli*) in groundwater poses health risks to humans, with health complications ranging from acute to chronic [26]. For instance, prevalences of diarrhoea, cholera, and dysentery associated with the consumption of microbially contaminated groundwater have been reported in the Limpopo province of South Africa [27,28,29]. Moreover, the occurrence of pollutants, such as pharmaceutical byproducts and heavy metals, tends to alter microorganisms’ genomes, consequently igniting the occurrence and spread of antimicrobial resistance and superbugs among water pathogens [30]. Samie et al. [31] have reported multiple resistant pathogens from boreholes in Giyani, Limpopo, South Africa. The occurrence of antimicrobial resistance can, consequently, exacerbate the challenge of high morbidity and mortality rates, as well as economic meltdowns globally [32]. Therefore, the surveillance of microbial pollution in groundwater can assist in identifying hotspots of antibiotic-resistant pathogens, guiding relevant interventions to mitigate their spread, and, consequently, safeguard human health.

The exposure of humans to heavy metals as well as pathogens in groundwater mainly happens through the consumption of polluted water [33]. Therefore, the oral route is often considered when evaluating the non-carcinogenic and/or carcinogenic health risks posed by heavy metals [34,35]. Moreover, the quantitative microbial health risk assessment (QMRA) is another tool employed to predict human health risks such as infection, illness, and/or death from exposure to pathogenic microorganisms [36]. The QMRA uses measurements of pathogens in the water as inputs to evaluate associated risks as outputs. The QMRA model has been widely used for bacterial risk assessments of water [37,38,39].

Mankweng is a township in the Capricorn District Municipality in the Limpopo province of South Africa with a population density of over 2800/km^2^. It consists of the University of Limpopo, Mankweng Hospital, clinics, shopping centres, filling stations, large human settlements, and agricultural farms, which rely heavily on water use. However, since Mankweng is a semi-arid township, the treated water supply from the municipality is inadequate; thus, some households, especially in new settlements, are not connected to the municipal line. Therefore, the unconnected households rely on groundwater from boreholes as an alternative source. Due to various activities and poor sanitation in some areas, groundwater may be contaminated with different physicochemical and microbial pollutants [40,41]. Moreover, the pollution is exacerbated by the unclear legislation and policies governing the use and quality of groundwater in South Africa. Therefore, while the South African Department of Water and Sanitation is still drafting a national groundwater strategy [42], it is of paramount importance to evaluate the quality of groundwater to protect the consumers. Although, to the best of our knowledge, there are no published data on heavy metal pollution in Mankweng township, there are few studies in the Limpopo province reporting on the heavy metal pollution in groundwater. However, there are scarce available data on microbiological pollutants and the potential health risks associated with both heavy metals and pathogens [40,43].

Therefore, our study focused on evaluating the heavy metal and bacterial pollution in groundwater from different households in Mankweng. Moreover, we quantitatively assessed the heavy metal pollution index and the potential health risks posed by exposure to heavy metals and pathogenic bacteria.

## 2. Materials and Methods

### 2.1. Study Area

**Figure 1:**
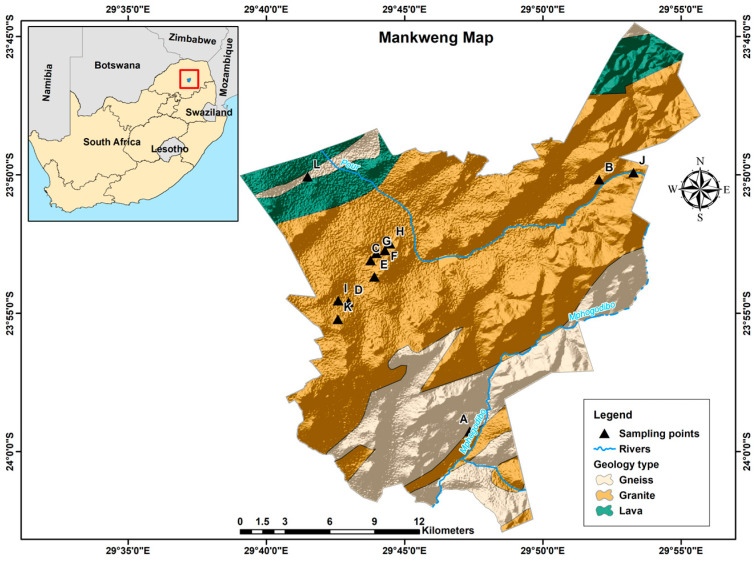
The location of South Africa and the topography of Mankweng township.

This study was conducted at Mankweng, a township within Capricorn District Municipality in the Limpopo province of South Africa. The area is located at corner coordinates 23°53′10″ S, 29°43′05″ E and 29°42′19.2″ E, 23°50′58.7″ S and is about 30 km east of Polokwane city. Mankweng covers a total area of 600 km^2^, with approximately 3,000,000 inhabitants. The area is characterised by hills (rocky outcrops, known as ‘koppies’) amidst mostly flat regions with elevations ranging from 1033 to 1876 m as shown in Figure 1. The predominant geological formations in the area consist of gneiss, granite, and lava. These rock types play a significant role in shaping the landscape and groundwater availability in the region.

### 2.2. Mankweng Climate

**Figure 2:**
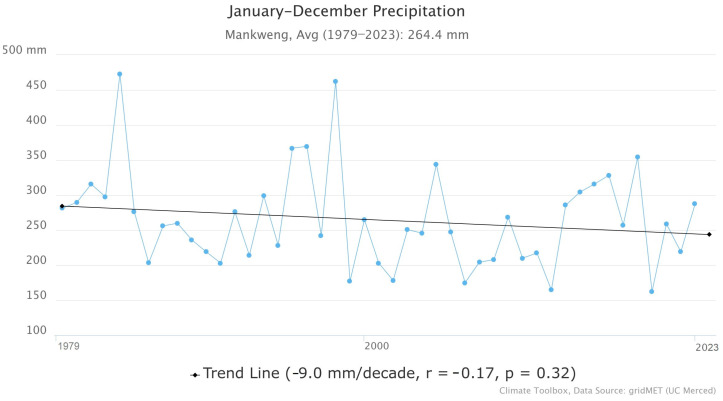
Precipitation at Mankweng.

Mankweng township has a semi-arid climate. According to the precipitation data from the Mankweng Meteorological Station shown in Figure 2, the monthly average precipitation for 1979–2023 ranged from 200 to 600 mm per annum [44]. The area receives its highest rainfall in summer (134 mm) from December to February and the lowest average rainfall (0 mm) in Winter, June, and July. In addition, there has been a slight downward trend in precipitation (Figure 2), which shows a decline of −9.0 mm per decade. Nonetheless, the *p*-value (0.32) showed that the trend was not statistically significant, and a weak correlation coefficient (*r* = −0.17) was also observed between time and precipitation. Despite the small downward trend, the rainfall in Mankweng remains highly variable year-to-year.

### 2.3. Sample Collection of Groundwater

This study was conducted during the winter season (June 2024), when there were no frequent rainfalls. The groundwater samples were collected from twelve (*n* = 12) residential boreholes, which were randomly selected. Factors such as distances of boreholes from septic tanks and latrines, the depths of the borehole, septic tanks and latrines, and diameters of boreholes that might contribute to groundwater pollution [45] were not considered during sampling. The groundwater samples were collected using the standard sampling procedures set by the American Public Health Association (APHA) 23rd Edition [46]. Before grab sampling, groundwater was flushed out from the boreholes for 2 min and was then used to rinse sterile Scott bottles twice. The collected samples were preserved on ice in a cooler box to maintain their integrity and to minimise degradation during transportation to the analytic laboratory at the University of Limpopo. Each sample was analysed for heavy metals, total coliforms (TC), and *E. coli*.

### 2.4. Detection of Heavy Metals 

The heavy metals designated as priority pollutants by the United States Environmental Protection Agency (USEPA) 23rd Edition were analysed in this study [47]. The concentrations of heavy metals such as copper (Cu), manganese (Mn), iron (Fe), chromium (Cr), cadmium (Cd), nickel (Ni), zinc (Zn), lead (Pb), vanadium (V), cobalt (Co), and aluminium (Al) were detected using inductively coupled plasma mass spectrophotometry (ICP-MS) (Perkin Elmer, Washington, DC, USA). ICP-MS is one of the cost-effective techniques used for compressive analysis of heavy metals from aquatic environments [48]. The instrument was standardised with a multi-element calibration standard IV for ICP for Cu, Mn, Fe, Cr, Cd, Ni, Zn, Pb, V, Co, and Al. The analytical precision was assessed by frequently analysing the standards and the blanks. The detection limit of each heavy metal was 0.01 mg·kg^−1^ [49].

### 2.5. Microbial Analysis

Bacterial isolation was conducted within 6 h of sample collection. TC and *E. coli* were quantified using the Colilert system. Briefly, the Colilert medium was added to 100 mL of the water sample and mixed until completely dissolved. Thereafter, the solutions were poured into an IDEXX Quanti-Tray/2000 and sealed using the Quanti-Tray sealer. Subsequently, the samples were incubated at 35 °C for 24 h. For quality assurance, the control blanks containing only quarter-strength Ringer’s solution were also prepared and incubated. After incubation, trays were observed to determine colour changes in the wells; yellow wells were indicative of the presence of the TC. All trays that were confirmed as TC were further examined under a fluorescent UV lamp at 365 nm. The wells that showed fluorescence under a UV lamp were considered to exhibit the presence of *E. coli*. The counts for both total coliforms and *E. coli* were determined using the most probable number (MPN) table, and the results were expressed as MPN/100 mL [50].

### 2.6. Quality Control

The background pollution of the groundwater samples was determined to ensure the accuracy of the generated data. The blanks were analysed after 4 samples, and the analyses were evaluated in triplicates. The precision and accuracy of the analysed heavy metals were assessed against standard references for each metal.

### 2.7. Determination of Heavy Metal Pollution Index

The groundwater quality was also evaluated using the heavy metal pollution index (HPI) method. In this analysis, the measurable limits for the standard permitted values (Si) and ideal values (Ii) were obtained from the WHO [4]. The HPI of groundwater was computed using Equation (1).

(1) $$HPI=\frac{\sum_{i=n}^{n}Wi\times Qn}{\sum_{i=n}^{n}Wi}$$

where W*_i_* denotes the unit weightage of the *i*th component; *n* is the number of selected parameters, and Q*n* is the subindex of the *i*th components. Q*n* is computed using the following equation:

(2) $$Qn=\sum_{i=n}^{n}\frac{\left(Mi-Ii\right)}{\left(Si-Ii\right)}\times 100,$$

where M*i* denotes the obtained monitored value (*i*th parameter in ppb), S*i* is the standard permissible value (*i*th parameter in ppb), and I*i* denotes the ideal value (ppb) *i*th parameter (minimum desirable value for drinking water). A value of HPI below 100 signifies non-contaminated groundwater, while a value above 100 implies contamination by heavy metals.

### 2.8. Non-Carcinogenic and Carcinogenic Health Risk Assessment

High concentrations of heavy metals beyond permissible levels are potential carcinogens to humans. Hence, in this study, we evaluated the non-carcinogenic and carcinogenic health risks of the detected heavy metal due to ingestion exposure. The United States Environmental Protection Agency (USEPA) guidelines were used to conduct the health risk assessment. 

#### 2.8.1. Non-Carcinogenic Health Risk Assessment

Non-carcinogenic risk refers to the potential for toxic effects caused by ingestion exposure to non-carcinogenic metals of concern. In this study, the non-carcinogenic health risk was evaluated utilising the chronic daily intake (CDI) and hazard quotient (HQ). The chronic daily intake was computed using Equation (3), as outlined by the US Environmental Protection Agency [51].

(3) $$CDI=\frac{Cgw\times IR\times EF\times ED}{AT\times BW},$$

where CDI denotes chronic daily intake through ingestion (mg/kg/day); Cgw: average concentration of the estimated metals in water (mg/L); IR: ingestion rate in this study (2.0 L/day for adults; 1.0 L/day for children); EF: exposure frequency (365 days/year); ED: exposure duration (70 years for adults; 10 years for children); AT: averaging time (25,550 days for an adult; 3650 days for a child); BW: average body weight (70 kg for adults; 30 kg for children) [52]. The different age groups underscore the relevance of age-specific considerations in health research and mitigation planning, as body weight variations can affect health outcomes. The hazard quotient (HQ) was calculated for non-carcinogenic risk using Equation (4).

(4) $$HQ=\frac{CDI}{RfD},$$

where RfD is the reference oral dose, representing the daily dosage that allows an individual to endure this level of exposure for an extended length of time without suffering any adverse effects. The RfD values (in mg/kg/day) used were Cd (1.00 × 10^−3^), Cr (3.00 × 10^−3^), Cu (3.7 × 10^−2^), Fe (0.7), Mn (1.4 × 10^−1^), Pb (3.60 × 10^−3^), Zn (3.0 × 10^−1^), and V (7 × 10^−3^). An HQ less than 1 indicates no adverse health impacts are expected due to exposure. Conversely, if the HQ is greater than 1, negative health effects are possible [53].

#### 2.8.2. Carcinogenic Health Risk Assessment

Cancer risk is the probability of developing cancer due to ingestion exposure to carcinogens. The International Agency for Research on Cancer (IARC) [54] classified Cd, Cr, and Pb as group 1 human carcinogens. Therefore, in this study, the human cancer risk of the detected carcinogens (Cd, Cr, and Pb) was evaluated. The carcinogenic health risk index (C) was used to determine the potential carcinogenic health risk of groundwater pollutants using Equation (5).

(5) C=CDI×SF

where SF is the slope coefficient. SF values (kg × day/mg) for Cd, Cr, and Pb used are 0.38, 0.5, and 8.5 × 10^−3^, respectively [53,54].

The cumulative carcinogenic health risk index (CT) of the detected carcinogens (Cd, Cr, and Pb) was calculated using the following formula:

CT = C_Cd_ + C_Cr_ + C_Pb_ (6)

C or CT values less than 1 × 10^−6^ were considered harmless, while C or CT values exceeding 1 × 10^−4^ were considered detrimental [55].

### 2.9. Quantitative Microbial Health Risk Assessment (QMRA)

Quantitative microbial risk assessment (QMRA) is an arithmetic modelling method used to predict the risk of infection and illness due to exposure to pathogens in the environment. QMRA analysis was used to determine the human health risk due to exposure of adults and children to pathogenic *E. coli* following consumption of the groundwater. The dose–response assessment was established based on the Beta-Poisson model, where the probability of infection per day (P*in*) was computed using Equation (7).

(7) $$Pin=1+{\left.\left(1+\frac{D}{\beta }\right.\right)}^{-\alpha },$$

where *D* refers to the average dose ingested obtained by multiplying the consumed volume of water per day by the recorded average value of *E. coli*. Based on the assumption that 8% of the total *E. coli* is pathogenic, the dose was further multiplied by 0.08 to predict the dose of pathogenic *E. coli* [36,56,57]. The average number of ingested *E. coli* was estimated by the mean concentration of *E. coli* and the volume of groundwater consumed by adults and children per day. The α (adult = 0.050 and children = 0.084) and *β* (adult = 1.001 and children = 1.44) were the dose–response parameters used for *E. coli* [56]. The annual risk of infection (P*an*) and the probability of illness per infection (P*il*) were also evaluated using Equations (8) and (9), respectively.

(8) $$Pan=1-({1-Pin)}^{n}$$

(9) $$Pil=\left[\left(Pan\times \frac{Pil}{Pin}\right)\right],$$

where *n* is the number of days of exposure per year (365 days) due to pathogenic *E. coli* dose, P*il* is the probability of illness, and $P\frac{il}{in}$ is the probability of illness per infection. $P\frac{il}{in}$ for *E. coli* was considered *as* 0.35 [58]. The calculated P*an* values were compared with the annual risk of infection limit for drinking water set by WHO, which restricts the infection cases to a maximum of 10^−4^ per person per year (pppy) [59].

### 2.10. Data Analysis

The experiments were conducted in triplicates and expressed in mean standard deviation. Data analysis was subjected to analysis of variance (ANOVA), and Tukey’s honestly significant difference test was used to determine the mean separation with a significant difference between the treatments indicated at *p* ≤ 0.05 using Graph Pad prism™ version 8.4.2. The Pearson correlation was performed using OriginPro 2024b software to analyse the relationship between the tested parameters. A value between 0 and 1 implied a positive correlation, while values between 0 and −1 signified a negative correlation between two variables at a significant level of *p* < 0.05. A zero value denotes no correlation between two parameters. A strong correlation is reflected when *r* > 0.7, whereas *r* between 0.5 and 0.7 indicates a positive moderate correlation [60]. Principal component analysis (PCA) was carried out to determine the potential sources of heavy metals using OriginPro 2024b software. The principal components with an eigenvalue > 1 were extracted. OriginPro 2024b software was also used to undertake a hierarchical cluster analysis (HCA) to analyse the generated data. Euclidean distance was used to determine the similarity between the selected sampling sites, and Ward’s method was employed as the joining rule.

## 3. Results

### 3.1. Heavy Metal Analysis of the Groundwater

Table 1 illustrates the heavy metal concentration in different boreholes. The metals Co, Ni, and Al were not detected in any of the groundwater samples, whereas Cd (0.002 mg/L) was only detected at site J. The concentration of Cr ranged from 0.002 to 0.013 mg/L, whereas the concentration of Cu ranged between 0.001 and 0.006 mg/L. Fe ranged from not detected to being detected in some groundwater samples. The highest Fe concentration of 0.025 mg/L was obtained at site J. Fe concentrations complied with the WHO [4] and SANS [3] standard of ≤2 mg/L. The detected Mn levels (0.001–0.019 mg/L) were within the acceptable limits of 0.08 and 0.4 mg/L by WHO [4] and SANS [3]. Pb concentrations ranged between 0.004 to 0.011 mg/L. Pb concentrations at sites E and G slightly exceeded the WHO [4] standard of ≤0.01 mg/L but were below the SANS [3] guideline of ≤0.10 mg/L. Zn was not detected in sites A, B, C, and H; however, the highest concentration of 0.008 mg/L was observed at site E. Zn concentrations complied with the SANS [3] and WHO [4] guidelines of ≤0.3 and 0.5 mg/L, respectively. V concentrations were in the range of 0.144 to 0.278 mg/L. The permissible guidelines of SANS [3] and WHO [4] for V concentrations for drinking water were not available. Generally, all the detected heavy metals in different sites, except Pb at sites E and G, were within acceptable limits according to the SANS [3] and WHO [4] standards.

### 3.2. Microbial Analysis of the Groundwater

The bacterial analysis of the groundwater samples was performed by detecting the presence of TC and *E. coli*. Based on the results, only site L was polluted with *E. coli* with a maximum count of 9 MPN/100 mL. The *E. coli* count was greater than the SANS [3] and WHO [4] guideline of <1 MPN/100 mL. Twenty-five percent (*n* = 3/12) of the groundwater sources were not contaminated with TC. However, samples from sites A and B had high pollution levels of TC, which were both greater than 201 MPN/100 mL. The TC count did not comply with the WHO [4] standard of <1 MPN/100 mL, except at sites D, G, and H. However, sites A, B, and L did not comply with the SANS [3] guideline of ≤10 MPN/100 mL for TC (Table 2).

### 3.3. Pearson Correlation Coefficients of Selected Groundwater Parameters

The relationships between the selected parameters are displayed shown in Table 3. The correlation analysis showed significantly strong positive correlations for Cd–Fe (*r* = 0.95), Cr–Pb (*r* = 0.72), and Mn–*E. coli* (*r* = 0.99). Moderate positive correlations (0.5 < *r* < 0.7) were observed for Mn–Cu (*r* = 0.56), Zn–Cu (*r* = 0.52), and EC–Cu (*r* = 0.57). On the other hand, moderate negative correlations were observed for Pb–Cd (*r* = −0.51), Mn–Pb (*r* = −0.51), and Mn–*E. coli* (*r* = −0.50).

### 3.4. PCA of the Parameters

Three main principal components (PCs) were selected based on their eigenvalues, which were greater than 1. As shown in Table 4, the three components explained 79.21% of the total variance extracted. PC 1 explained 36.41%; PC 2, 25.60%; and PC 3, 17.20%.

### 3.5. Bioplot of the Main PCs

**Figure 3:**
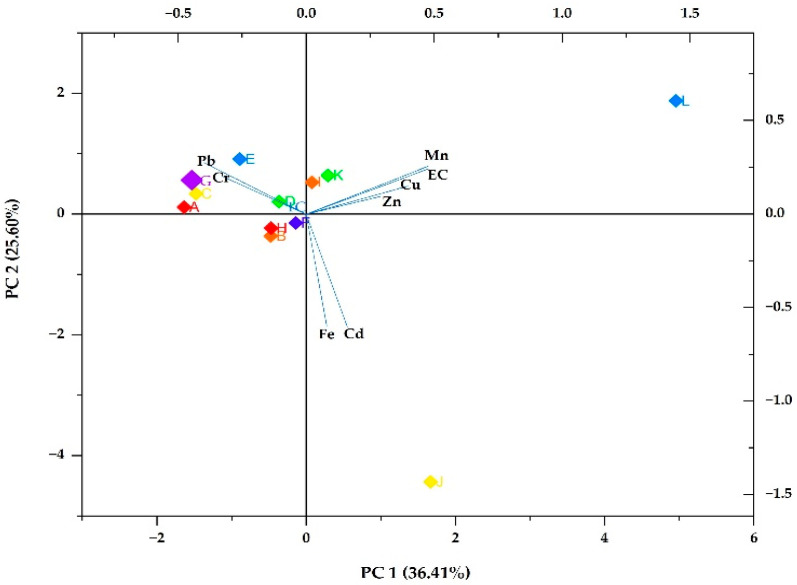
Bioplot of the main two PCs. EC and TC denote total coliforms and *E. coli*, respectively. The coloured boxes demonstrate the sampling sites.

PCA was utilised to identify the main source of pollution. Figure 3 demonstrates a biplot of PC 1 and PC 2. PC 1 exhibited the highest low positive loading for Mn (0.47733) and *E. coli* (0.4763). PC 1 also revealed weak negative loading for Pb (−0.40005) and Cr (−0.33036). PC 2 revealed the highest weak positive loading of Pb (0.2759), *E. coli* (0.25628), Mn (0.24248), and Cr (0.20305). It also revealed moderate negative loading for Cd (−0.60548) and Fe (−0.59962).

### 3.6. HCA of the Sampling Sites

**Figure 4:**
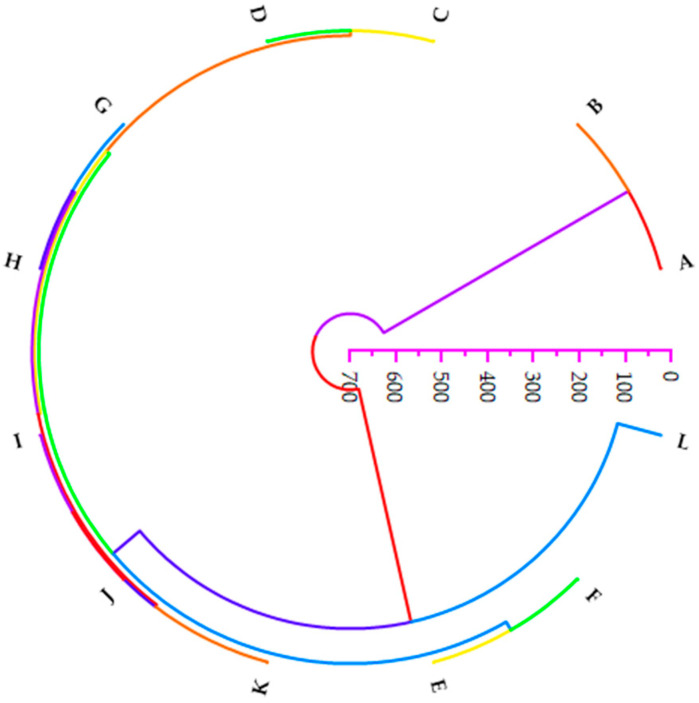
Dendrogram illustrating the clustering of sampling sites of the groundwater from Mankweng.

The dendrogram illustrating the relationship between the sampled points is shown in Figure 4. There were three clusters produced, of which Cluster 1 was composed of sites A and B and Cluster 3 included only site L. The rest of the sites were classified in Cluster 2, with some subclusters. As seen from the dendrograms, the sites in Cluster 1 had the closest distance. Regarding the distance between clusters, clusters 1 and 2 were closer to each other and differed more from Cluster 3.

### 3.7. HPI of the Groundwater

HPI was arithmetically evaluated, and the results are displayed in Table 5. HPI values obtained in this study were all under the maximum permissible HPI value of 100. The highest HPI value of 87.338 was obtained at site H, whereas the lowest (66.187) was at site I.

### 3.8. Quantitative Heavy Metal Health Risk Assessment

#### 3.8.1. Chronic Daily Intake (CDI)

The health risk assessments were not performed for Co, Ni, and Al as their concentrations were below the detection. The health risk assessment was assessed by evaluating the CDI through the ingestion route, and the findings are illustrated in Table S1. Based on the findings in this study, the CDIs of the detected metals for both adults and children were found in the order of Fe < Zn < Mn < Cu < Cr < Cd < Pb < V. Moreover, only V CDI values at site I (adult = 7.71 × 10^−3^ and children = 9.0 × 10^−3^) and site K (adult = 7.94 × 10^−3^ and children = 9.27 × 10^−3^) exceeded the corresponding V RfD value used in this study, while the CDIs of other metals were lower than the RfD values. The magnitude of risk due to exposure through groundwater consumption was observed to be higher for children than for adults for all metals.

#### 3.8.2. Hazard Quotient (HQ) of the Heavy Metals

Table S2 illustrates the non-carcinogenic risk estimates for Cd, Cr, Cu, Fe, Mn, Pb, Zn, and V. The HQ values of the detected heavy metals were all less than 1 in both the adults and children age groups except for V, which was found to be higher than 1 at site I (adult = 1.10 and children = 1.286) and site K (adult = 1.13 and children = 1.324). The HQ ingestion for adults was in the ranges of 1.280–10.560, 0.107–0.853, 0.013–0.049, 0.098–0.239, 0.061–0.107, 0–0.128, and 0.137–0.434 for Cd, Cr, Cu, Fe, Mn, Pb, Zn, and V, respectively. The HQ ingestion values for children were in the ranges of 0.107–10.453, 0.107–0.747, 0.011–0.034, 0.089–0.236, 0.056–0.107, 0.000–0.064, and 0.091–0.206 for Cd, Cr, Cu, Fe, Mn, Pb, Zn, and V, respectively. Moreover, HQ values were generally all higher for children in comparison to those of adults.

### 3.9. Carcinogenic Health Risk

Table S3 illustrates the probability of cancer risk of the three carcinogenic heavy metals (Cr, Cd, and Pb) for both adults and children. The mean Cd for children at site J was 2.17 × 10^−5^ for adults and 2.53 × 10^−5^ for children. The Cd records fell within the acceptable range of 1 × 10^−6^ to 1 × 10^−4^. The mean Cr ranged between 2.86 × 10^−5^ and 1.86 × 10^−4^ for adults and 3.34 × 10^−5^ and 2.17 × 10^−4^ for children. The highest cancer risk for children was, therefore, up to two times higher than the acceptable limit of 1.0 × 10^−4^. The mean Pb was in the range of 1.95 × 10^−6^ to 2.67 × 10^−6^ for adults and 2.09 × 10^−6^ to 3.13 × 10^−6^ for children and were higher than the acceptable limit of 1.0 × 10^−6^. Based on the acceptable range for cancer risk (≤1 × 10^−6^ to 1 × 10^−4^), 67 and 58% of the sampled sites had negligible cumulative cancer risk for adults and children due to Cr, Cd, and Pb contents. The cumulative carcinogenic risk for children was generally higher compared to adults at all sites.

### 3.10. Quantitative Microbial Health Risk Assessment

The presence of *E. coli* was only confirmed at borehole water from site L; therefore, the risks of infection and illness through ingestion of this pathogen upon water consumption were evaluated. The values calculated for the risk of infection per day (P*in*) were 2.7 × 10^−2^ for adults and 3.3 × 10^−2^ for children. The annual risk of infection (P*an*) for adults was 7.56 × 10^−1^ and 8.3 × 10^−1^. The risks of illness (P*il*) for adults and children were 2.65 × 10^−1^ and 2.91 × 10^−1^, respectively.

## 4. Discussion

The exposure of humans to heavy metal pollution is a major concern, particularly in developing countries. The pollution is mainly due to anthropogenic activities and the geological properties of underground stones, which consequently pose health-related risks [61]. The lack of information might be due to the general misconception that groundwater is free from pollutants such as heavy metals and pathogens, or the high analytical costs involved.

In this study, the heavy metal and bacteriological pollution of groundwater were evaluated. The undetected Co, Ni, and Al might be due to the detection limits of the instrument used since the presence of these heavy metals has been previously reported in groundwater in some studies conducted in the Limpopo province [62,63,64,65].

The permissible guidelines of SANS [3] and WHO [4] for V concentrations for drinking water were not available. Nevertheless, all the detected heavy metals (Cd, Cr, Cu, Fe, Mn, and Zn) were within the recommended SANS [3] and WHO [4] limits, except Pb levels at sites E and G, which did not comply with the acceptable limit according to WHO [4] guidelines. However, based on the SANS [3] standard of ≤ 0.10 mg/L, Pb levels complied at all sites including sites E and G. According to WHO [4] guidelines, the high concentration of Pb at sites E and G meant that consumers of the groundwater are at a high health risk of its toxic effects. The sites E and G are newly constructed boreholes at private student accommodations around the University of Limpopo campus. Therefore, the high Pb levels at these sites might be due to anthropogenic activities such as dumping of Pb-rich waste near constructed boreholes, which consequently might infiltrate into the groundwater. Pb is linked with harmful effects on multiple human organs. It is particularly harmful to brain cells, resulting in mental retardation and dementia. Furthermore, Pb exposure can result in anaemia, renal impairment, hypertension, and reproductive organ dysfunction [66]. The findings in this study were similar to those of Dube et al. [67], who reported Pb values from the Mokopane District of Limpopo province as falling within the SANS [3] recommendations but being higher than the WHO [4] standards. Therefore, appropriate groundwater treatment at these sites is needed to safeguard consumers’ health.

*E. coli* contamination only exceeded the SANS [3] and WHO [4] at site L, implying the groundwater to be suitable for consumption at all sites, except at site L, where it was deemed unfit for consumption. Moreover, site L also revealed high TC concentration, further testifying to the health threat to consumers. The high bacterial pollution at site L might be from the poorly treated wastewater from the nearby wastewater treatment plant, which supplies wastewater to this site for irrigation and farm animal drinking purposes (e.g., for cows, goats, and sheep). Moreover, the leaching of these bacteria from animal waste into the boreholes might also be the main contributor to microbial pollution at this site [68]. Kgopa et al. [40] previously conducted the microbial analysis of groundwater at site L and found the water to be polluted with diverse pathogenic microorganisms. The high concentration of TC beyond the SANS [3] and WHO [4] limit standards at some sampled sites might be attributed to the faecal matter from pit latrines and septic tanks constructed within 15–30 m from the constructed boreholes [45,69]. The presence of TC and *E. coli* beyond SANS [3] and WHO [4] limits for drinking water poses a health threat to consumers [70]. Therefore, appropriate groundwater treatments such as boiling are needed at the affected sites before drinking to safeguard consumers’ health against infections and illnesses due to exposure to pathogens.

Pearson correlation and PCA were used to determine possible sources of heavy metals and microbial pollutants in the groundwater. PC 1 was dominated by Mn, *E. coli*, and Cu, of which Mn-*E. coli* were observed to have a strong significant correlation, implying that Mn can be used to predict the prevalence of *E. coli* in the groundwater. The observation meant that the higher the Mn concentration in water, the higher the *E. coli* presence in groundwater. Moreover, Mn and Cu revealed a moderate positive correlation, implying that these metals originate from the same source. The Mn concentration was well below the baseline set by SANS [3] and WHO [4], suggesting that its content might be impacted by natural sources rather than human activities. The maximum Mn content at site L was attributed to agricultural activities such as the application of manure and fertilisers. Pb and Cr also revealed a substantial loading on PC 1 and a significantly strong negative correlation, indicating the elements to be the dominant pollutants. The low negative loading by Pb, with some values exceeding the safe water criteria set by WHO [4] at some sites, suggested that the presence of Pb can be related to anthropogenic activities like the application of fertilisers containing Pb. However, the negative loading implied that Pb did not influence other parameters. In PC 2, Cd and Fe were the dominant pollutants; they were also found to have a significant positive correlation, suggesting these elements originate from the same source.

Hierarchical cluster analysis was employed to understand the relationship between the sampling points. The sampling sites close to each other revealed similar characteristics. Moreover, as illustrated by the dendrograms, the sites in Cluster 1 had the closest distance, indicative of the highest similarity. PCA and HCA agreed with each other and showed the significant contributions and sources of these metals in groundwater samples.

The HPI was utilised to identify possible effects of heavy metal pollution. The HPI values obtained in this study were all under the maximum permissible HPI value of 100, implying that the metals in groundwater represented a negligible level of pollution that may not cause adverse effects on the health of Mankweng residents [13]. The results correlate with the findings of Lotfi et al. [71], whereby the HPI values were all below the maximum permissible HPI value in all sites.

The RfD value represents an estimated value of the daily exposure to metals that does not have a hazardous effect on humans during a lifetime [72]. The V CDI values exceeding the RfD value in this study implied that V poses a serious health threat to the groundwater consumers in the Mankweng area. This meant that groundwater consumers were at a high risk of suffering from stomach cramps, nausea, diarrhoea, and, in certain cases, lung cancer due to exposure to V [73,74]. Similar findings were observed in the Eastern Cape province of South Africa by Mandindi et al. [75], whereby the CDI value of V was greater than the RfD value, indicative of health risk. However, the findings by John et al. [76] contrasted our observation as the CDI of V was well below the RfD value, suggesting V poses a negligible health risk. The CID values of the other metals in this study were well below the RfD values, signifying low or no health risk upon oral exposure route. The HQ values of the detected heavy metals were all less than 1 in both the adult and child age groups except for V, which was found to be higher than 1 at sites I and K, indicating that there are no potential non-carcinogenic health risks upon consumption of the groundwater. However, only V is linked to a possible non-carcinogenic effect upon consumption as it revealed an HQ value higher than 1. This implied that residents of Mankweng, especially residents of sites I and K, may develop non-cancerous disorders due to the high values of HQ for V. The high V HQ values observed at sites I and K raise health concerns because V is a recognised neurotoxicant that can interfere with critical physiological and biochemical processes in the central nervous system. It is also known to be immunotoxic and to induce the production of reactive oxygen species, which can be detrimental to humans [73,77].

The International Agency for Research on Cancer (IARC) has classified carcinogenic pollutants based on their carcinogenicity to humans and animals. Carcinogenic risk defines the probability of a population acquiring cancer because of the ingestion of carcinogens [78]. Based on the recommendations of USEPA, the carcinogenic risk range for carcinogens is ≤1 × 10^−6^ to 1 × 10^−4^ [55]. Therefore, based on the standard, there is a low cancer risk due to Cd and Pb contents in groundwater from Mankweng. However, some sites pose carcinogenic health threats both to adults and children as the cancer risk values for Cr exceeded the set guideline. The findings were comparable to those of Edokpayi et al. [5], who reported that the carcinogenic exposure risk for Cr in the groundwater exceeded the limits. However, their findings on Pb cancer risk values, which exceeded the standards, contradicted our results.

The acceptable annual risk of infection for the waterborne pathogen is 10^−4^ pppy [59]. In this study, the obtained annual risk of infection values for both the adults and children exceeded the WHO [4] acceptable risk limit, indicating that the health risk is probable to occur following exposure to *E. coli* through groundwater consumption. Moreover, this may lead to the transmission of this pathogen to the environment and the possible occurrence of resistant strains [79]. The risk of illness values for adults and children were high, suggesting the possible high prevalence of illnesses associated with *E. coli* in the area [14]. Therefore, *E. coli* poses a major threat to the health of groundwater consumers at Site L. The results suggest the need to treat the groundwater from this borehole for *E. coli* contamination through techniques such as boiling before consumption [80]. The findings correlated with those obtained by Odiyo et al. [81] in Vhuronga 1, Limpopo province, South Africa, whereby *E. coli* revealed high risks of infection and illness per year.

There were some limitations in our study which included sampling once and not monthly or seasonally, whereas water quality may change over time. Moreover, factors such as distances of boreholes from septic tanks and latrines, the depths of the borehole, septic tanks and latrines, and diameters of boreholes were not considered during sampling. We only studied *E. coli*, based on resource availability; however, other pathogenic coliforms can be considered in the future. Lastly, the predictions of pathogenic *E. coli* were based on assumed doses, using estimates from the literature and the obtained *E. coli* counts from site L only. However, despite these limitations, the results in this study are relevant and underscored the need for regular monitoring and proper maintenance of groundwater from boreholes to safeguard human health. Moreover, this study gives a glimpse of the level of metal and bacterial pollution in groundwater from some boreholes, which might need serious remedial attention.

## 5. Conclusions

This study underscores the need to monitor heavy metal and microbial pollution in groundwater to safeguard human health. In the present study, all the detected heavy metals at different sites, except Pb at sites E and G, were within acceptable limits according to SANS and WHO standards. The HPI values of the metals were all under the maximum permissible levels, suggesting a negligible level of pollution that may not cause adverse effects on the health of consumers. The presence of TC and *E. coli* beyond SANS and WHO guidelines at some sites implied that the groundwater was unfit for consumption. The CID and HQ values of all the detected heavy metals, except for those of V, demonstrated the metals to pose no health risk upon oral exposure route. The cumulative carcinogenic risk values indicated potential adverse effects at some sampled sites. The bacterial risks of infection and illness values for adults and children at site L were higher than the acceptable guidelines, indicating that the health risks were probable to occur following exposure to *E. coli* through consumption of the groundwater. Most of the groundwater was safe for drinking due to the low presence of some heavy metals and the absence of *E. coli*. However, it is recommended that the consumers of the identified polluted groundwater adopt the point-of-use treatment before drinking the groundwater to safeguard their health. For future studies, the evaluation of the correlation between the prevalence of heavy metal pollution and antimicrobial resistance is important.

## Figures and Tables

**Table 1:** Heavy metal concentrations in the groundwater from Mankweng.

Parameter (mg/L)	WHO Std	SANS Std	Site												
			A	B	C	D	E	F	G	H	I	J	K	L	Average
Cd	≤0.003	≤0.003	ND	ND	ND	ND	ND	ND	ND	ND	ND	0.002 ± 0.006	ND	ND	0.002 ± 0.006
Cr	≤0.05	≤0.05	0.011 ± 001 ^c,d^	0.002 ± 0.000 ^a^	0.01 ± 0.001 ^c^	0.007 ± 0.002 ^b^	0.013 ± 0.001 ^d^	0.002 ± 0.000 ^a^	0.011 ± 0.001 ^c,d^	0.002 ± 0.000 ^a^	0.004 ± 0.001 ^a^	0.002 ± 0.001 ^a^	0.004 ± 0.000 ^a^	0.002 ± 0.000 ^a^	0.006 ± 0.004
Co	≤0.05	≤0.5	ND	ND	ND	ND	ND	ND	ND	ND	ND	ND	ND	ND	ND
Cu	≤2	≤2	0.001 ± 0.000 ^a^	0.001 ± 0.000 ^a^	0.001 ± 0.000 ^a^	0.002 ± 0.001 ^a,b^	0.003 ± 0.002 ^a,b^	0.001 ± 0.000 ^a^	0.001 ± 0.000 ^a^	0.001 ± 0.000 ^a^	0.004 ± 0.000 ^a,b^	0.003 ± 0.000 ^a,b^	0.006 ± 0.001 ^c^	0.006 ± 0.002 ^c^	0.003 ± 0.002
Fe	≤2	≤2	0.005 ± 0.002 ^a,b^	0.003 ± 0.001 ^a,b^	0.001 ± 0.000 ^a,b^	ND	0.004 ± 0.002 ^a^	0.005 ± 0.002 ^a^	0.004 ± 0.001 ^a^	ND	ND	0.025 ± 0.002 ^b^	ND	ND	0.007 ± 0.008
Mn	≤0.08	≤0.4	0.001 ± 0.005 ^a,b^	0.002 ± 0.001 ^a^	0.001 ± 0.000 ^a^	0.003 ± 0.002 ^a^	0.001 ± 0.000 ^a^	0.002 ± 0.001 ^a^	0.003 ± 0.003 ^a,b^	0.001 ± 0.000 ^a^	0.001 ± 0.000 ^a^	0.002 ± 0.001 ^a^	0.002 ± 0.000 ^a^	0.019 ± 0.018 ^b^	0.003 ± 0.005
Ni	≤0.02	≤0.07	ND	ND	ND	ND	ND	ND	ND	ND	ND	ND	ND	ND	ND
Pb	≤0.01	≤0.10	0.01 ± 0.004 ^a^	0.009 ± 0.00 ^a^	0.01 ± 0.001 ^a^	0.009 ± 0.000 ^a^	0.011 ± 0.003 ^a^	0.01 ± 0.003 ^a^	0.011 ± 0.004 ^a^	0.009 ± 0.000 ^a^	0.01 ± 0.002 ^a^	0.008 ± 0.000 ^a^	0.01 ± 0.001 ^a^	0.008 ± 0.000 ^a^	0.01 ± 0.001
Zn	≤3.0	≤5	ND	ND	ND	0.001 ± 0.000 ^a^	0.008 ± 0.001 ^a^	0.006 ± 0.001 ^a^	0.002 ± 0.001 ^a^	ND	0.005 ± 0.000 ^a^	0.004 ± 0.000 ^a^	0.002 ± 0.001 ^a^	0.007 ± 0.002 ^a^	0.004 ± 0.002
Al	≤0.9	–	ND	ND	ND	ND	ND	ND	ND	ND	ND	ND	ND	ND	ND
V	–	–	0.183 ± 0.004 ^c^	0.16 ± 0.015 ^a,b^	0.184 ± 0.007 ^c^	0.181 ± 0.008 ^c^	0.176 ± 0.002 ^b,c^	0.172 ± 0.004 ^b,c^	0.183 ± 0.003 ^c^	0.167 ± 0.007 ^a,b,c^	0.27 ± 0.015 ^d^	0.144 ± 0.003 ^a^	0.278 ± 0.008 ^d^	0.189 ± 0.001 ^c^	0.191 ± 0.04

The superscripts (a, b, c, and d) represent statistical differences at *p* < 0.05.

**Table 2:** Total coliforms and *E. coli* concentrations (MPN/100 mL) of the groundwater.

Site	*E. coli*	TC
A	<1	>201
B	<1	>201
C	<1	1
D	<1	<1
E	<1	10
F	<1	9
G	<1	<1
H	<1	<1
I	<1	2
J	<1	3
K	<1	2
L	9	59
SANS [3]	<1	≤10
WHO [4]	<1	<1

**Table 3:** Pearson correlation coefficient of the selected parameters.

	Cd	Cr	Cu	Fe	Mn	Pb	Zn	Total Coliforms	*E. coli*
Cd	1								
Cr	−0.28	1							
Cu	0.08	−0.28	1						
Fe	0.95 *	−0.14	−0.08	1					
Mn	−0.07	−0.29	0.56	−0.16	1				
Pb	−0.50	0.72 *	−0.26	−0.33	−0.51	1			
Zn	0.11	−0.07	0.52	0.14	0.41	0.05	1		
Total coliforms	−0.15	0.02	−0.24	−0.02	0.05	−0.13	−0.34	1	
*E. coli*	−0.09	−0.28	0.57	−0.18	0.99 *	−0.50	0.43	0.08	1

The * denotes Pearson correlation is significant at *p* < 0.05.

**Table 4:** PCA of the evaluated selected parameters.

PC	Eigenvalue	Percentage of Variance (%)	Cumulative (%)
1	3.27674	36.41	36.41
2	2.30418	25.60	62.01
3	1.54795	17.20	79.21
4	0.87805	9.76	88.97
5	0.47516	5.28	94.25
6	0.4183	4.65	98.89
7	0.08717	0.97	99.86
8	0.00999	0.11	99.97
9	0.00247	0.03	100

**Table 5:** HPI of the groundwater from different sites.

Parameter	WnQn											
	Site											
	A	B	C	D	E	F	G	G	H	I	G	K
Cd (mg/L)	ND	ND	ND	ND	ND	ND	ND	ND	ND	45.382	ND	0.163
Cr (mg/L)	0.899	0.163	0.817	0.572	1.062	0.163	0.899	0.163	0.327	0.163	0.327	ND
Cu (mg/L)	5.11 × 10^−5^	5.11 × 10^−5^	5.11 × 10^−5^	0.000	0.000	5.106 × 10^−5^	5.11 × 10^−5^	5.11 × 10^−5^	0.00	0.000	0.000	0.000
Fe (mg/L)	0.000	0.000	5.11 × 10^−5^	ND	0.000	0.000	0.000	ND	ND	0.001	ND	ND
Mn (mg/L)	1.532	1.361	1.532	1.191	1.532	1.361	1.191	1.532	1.532	1.361	1.361	1.532
Pb (mg/L)	20.422	18.380	20.422	18.380	22.464	20.422	22.464	18.380	20.422	16.338	20.422	16.338
Zn (mg/L)	ND	ND	ND	0.000	4.55 × 10^−5^	9.11 × 10^−5^	0.000	ND	0.000	0.000	0.000	6.83 × 10^−5^
V (mg/L)	3.737	3.268	3.758	3.696	3.594	3.513	3.737	3.410	5.514	2.941	5.677	3.860
HPI	83.463	72.736	83.270	74.910	89.745	79.745	88.615	73.955	87.338	66.187	87.317	68.793

## Data Availability

Data are contained within the manuscript and Supplementary Materials. Further inquiries can be directed to the corresponding authors.

## Supplementary Materials

The following supporting information can be downloaded at https://www.mdpi.com/article/10.3390/ijerph21111489/s1: Table S1. CDI values of heavy metals detected in groundwater in Mankweng; Table S2. HQ values of selected metals detected in groundwater; Table S3. Carcinogenic health risk of heavy metals from groundwater samples in Mankweng.
