# Supplementary material for: Quantitative Assessment of Human Health Risks Associated with Heavy Metal and Bacterial Pollution in Groundwater from Mankweng in Limpopo Province, South Africa
Source: Int J Environ Res Public Health. 2024 Nov 9;21(11):1489. doi: 10.3390/ijerph21111489 (PMC11594182; doi:10.3390/ijerph21111489)
Supplement: Supplementary file 1 [file ijerph-21-01489-s001.zip › ijerph-3231102-supplementary.pdf]

International Journal of
Environmental Research and Public Health

MDPI

Table S1. CDI values of heavy metals detected in groundwater in Mankweng.

Site	Individual	CDI_{ing} (mg/kg/day)							
		**Cd**	**Cr**	**Cu**	**Fe**	**Mn**	**Pb**	**Zn**	**V**
A	Adult	–	3.14×10^{-4}	2.86×10^{-5}	1.43×10^{-4}	2.86×10^{-5}	2.86×10^{-4}	–	5.23×10^{-3}
	Child	–	3.67×10^{-4}	3.33×10^{-5}	1.67×10^{-4}	3.33×10^{-5}	3.33×10^{-4}	–	6.1×10^{-3}
B	Adult	–	5.71×10^{-5}	2.86×10^{-5}	8.57×10^{-5}	5.71×10^{-5}	2.57×10^{-4}	–	4.57×10^{-3}
	Child	–	6.67×10^{-5}	3.33×10^{-5}	1.0×10^{-4}	6.67×10^{-5}	3.0×10^{-4}	–	5.33×10^{-3}
C	Adult	–	2.86×10^{-4}	2.86×10^{-5}	2.86×10^{-5}	2.86×10^{-5}	2.86×10^{-4}	–	5.26×10^{-3}
	Child	–	3.33×10^{-4}	3.33×10^{-5}	3.33×10^{-5}	3.33×10^{-5}	3.33×10^{-4}	–	6.13×10^{-3}
D	Adult	–	2.0×10^{-4}	5.71×10^{-5}	–	8.57×10^{-5}	2.57×10^{-4}	2.86×10^{-5}	5.17×10^{-3}
	Child	–	2.33×10^{-4}	6.67×10^{-5}	–	1.0×10^{-4}	3.0×10^{-4}	3.33×10^{-5}	6.03×10^{-3}
E	Adult	–	3.71×10^{-4}	8.57×10^{-5}	1.14×10^{-4}	2.86×10^{-5}	3.14×10^{-4}	2.29×10^{-4}	5.03×10^{-3}
	Child	–	4.33×10^{-4}	1.0×10^{-4}	1.33×10^{-4}	3.33×10^{-5}	3.67×10^{-4}	2.67×10^{-4}	5.87×10^{-3}
F	Adult	–	5.71×10^{-5}	2.86×10^{-5}	2.86×10^{-5}	5.71×10^{-5}	2.86×10^{-4}	1.71×10^{-4}	4.91×10^{-3}
	Child	–	6.67×10^{-5}	3.33×10^{-5}	3.33×10^{-5}	6.67×10^{-5}	3.33×10^{-4}	2.0×10^{-4}	5.73×10^{-3}
G	Adult	–	3.14×10^{-4}	2.86×10^{-5}	1.14×10^{-4}	8.57×10^{-5}	3.14×10^{-4}	5.71×10^{-5}	5.23×10^{-3}
	Child	–	3.67×10^{-4}	3.33×10^{-5}	1.33×10^{-4}	1.0×10^{-4}	3.67×10^{-4}	6.67×10^{-5}	6.1×10^{-3}
H	Adult	–	5.71×10^{-5}	2.86×10^{-5}	–	2.86×10^{-5}	2.57×10^{-4}	–	4.77×10^{-3}
	Child	–	6.67×10^{-5}	3.33×10^{-5}	–	3.33×10^{-5}	3.0×10^{-4}	–	5.57×10^{-3}
I	Adult	–	1.14×10^{-4}	1.14×10^{-4}	–	2.86×10^{-5}	2.86×10^{-4}	1.43×10^{-4}	7.71×10^{-3}
	Child	–	1.33×10^{-4}	1.33×10^{-4}	–	3.33×10^{-5}	3.33×10^{-4}	1.67×10^{-4}	9.0×10^{-3}
J	Adult	5.71×10^{-5}	5.71×10^{-5}	8.57×10^{-5}	7.14×10^{-4}	5.71×10^{-5}	2.29×10^{-4}	1.14×10^{-4}	4.11×10^{-3}
	Child	6.67×10^{-5}	6.67×10^{-5}	1.0×10^{-4}	8.33×10^{-4}	6.67×10^{-5}	2.46×10^{-4}	1.33×10^{-4}	4.8×10^{-3}
K	Adult	–	1.14×10^{-4}	1.71×10^{-4}	–	5.71×10^{-5}	2.86×10^{-4}	5.71×10^{-5}	7.94×10^{-3}
	Child	–	1.33×10^{-4}	2.0×10^{-4}	–	6.67×10^{-5}	3.33×10^{-4}	6.67×10^{-5}	9.27×10^{-3}
L	Adult	–	5.71×10^{-5}	1.71×10^{-4}	–	5.42×10^{-4}	2.29×10^{-4}	2.0×10^{-4}	5.40×10^{-3}
	Child	–	6.67×10^{-5}	2.0×10^{-4}	–	6.44×10^{-4}	2.46×10^{-4}	2.33×10^{-4}	6.3×10^{-3}

Table S2. HQ values of selected metals detected in groundwater.

Site	Individual	Cd	Cr	Cu	Fe	Mn	Pb	Zn	V
A	Adult	–	1.05 x10^{-1}	7.73 x 10^{-4}	2.04 x 10^{-4}	2.04 x 10^{-4}	7.94 x 10^{-2}	–	7.47 x 10^{-1}
	Child	–	1.22 x 10^{-1}	9.0 x 10^{-4}	2.39 x 10^{-4}	2.38 x 10^{-4}	9.25 x 10^{-2}	–	8.71 x 10^{-1}
B	Adult	–	1.90 x 10^{-2}	7.73 x 10^{-4}	1.22 x 10^{-4}	4.08 x 10^{-4}	7.13 x 10^{-2}	–	6.53 x 10^{-1}
	Child	–	2.22 x 10^{-2}	9.0 x 10^{-4}	1.43 x 10^{-4}	4.76 x 10^{-4}	8.33 x 10^{-2}	–	7.61 x 10^{-1}
C	Adult	–	9.53 x 10^{-2}	7.73 x 10^{-4}	4.09 x 10^{-5}	2.04 x 10^{-4}	7.94 x 10^{-2}	–	7.51 x 10^{-1}
	Child	–	1.11 x 10^{-1}	9.0 x 10^{-4}	4.76 x 10^{-5}	2.38 x 10^{-4}	9.25 x 10^{-2}	–	8.76 x 10^{-1}
D	Adult	–	6.67 x 10^{-2}	1.54 x 10^{-3}	–	4.29 x 10^{-4}	7.13 x 10^{-2}	9.53 x 10^{-5}	7.39 x 10^{-1}
	Child	–	7.77 x 10^{-2}	1.80 x 10^{-3}	–	7.14 x 10^{-4}	8.33 x 10^{-2}	1.11 x 10^{-4}	8.61 x 10^{-1}
E	Adult	–	1.24 x 10^{-1}	2.31 x 10^{-3}	1.63 x 10^{-4}	2.04 x 10^{-4}	8.72 x 10^{-2}	7.63 x 10^{-4}	7.19 x 10^{-1}
	Child	–	1.44 x 10^{-1}	2.70 x 10^{-3}	1.9 x 10^{-4}	2.38 x 10^{-4}	1.02 x 10^{-1}	8.9 x 10^{-4}	8.39 x 10^{-1}
F	Adult	–	1.90 x 10^{-2}	7.73 x 10^{-4}	4.09 x 10^{-5}	4.08 x 10^{-4}	7.94 x 10^{-2}	5.70 x 10^{-4}	7.01 x 10^{-1}
	Child	–	2.22 x 10^{-2}	9.0 x 10^{-4}	4.76 x 10^{-5}	4.76 x 10^{-4}	9.25 x 10^{-2}	6.66 x 10^{-4}	8.19 x10^{-1}
G	Adult	–	1.05 x 10^{-1}	7.73 x 10^{-4}	1.63 x 10^{-4}	4.29 x 10^{-4}	8.72 x 10^{-2}	1.90 x 10^{-4}	7.47 x 10^{-1}
	Child	–	1.22 x 10^{-1}	9.0 x 10^{-4}	1.9 x 10^{-4}	7.14 x 10^{-4}	1.02^{-1}	2.22 x 10^{-4}	8.71 x 10^{-1}
H	Adult	–	1.90 x 10^{-2}	7.73 x 10^{-4}	–	2.04 x 10^{-4}	7.13 x 10^{-2}	–	6.81 x 10^{-1}
	Child	–	2.22 x 10^{-2}	9.0 x 10^{-4}	–	2.38 x 10^{-4}	8.33 x 10^{-2}	–	7.96 x 10^{-1}
I	Adult	–	3.8 x 10^{-2}	3.08 x 10^{-3}	–	2.04 x 10^{-4}	7.94 x 10^{-2}	4.77 x 10^{-4}	1.10
	Child	–	4.43 x 10^{-2}	3.59 x 10^{-3}	–	2.38 x 10^{-4}	9.25 x 10^{-2}	5.56 x 10^{-4}	1.286
J	Adult	5.17 x 10^{-2}	1.90 x 10^{-2}	2.31 x 10^{-3}	1.02 x 10^{-3}	4.08 x 10^{-4}	6.63 x 10^{-2}	3.8 x 10^{-4}	0.587
	Child	6.67 x 10^{-2}	2.22 x 10^{-2}	2.70 x 10^{-3}	1.19 x 10^{-3}	4.76 x 10^{-4}	1.02 x 10^{-1}	4.43 x 10^{-4}	6.86 x 10^{-1}
K	Adult	–	3.8 x 10^{-2}	4.62 x10^{-3}	–	4.08 x 10^{-4}	7.94 x 10^{-2}	1.90 x 10^{-4}	1.13
	Child	–	4.43 x 10^{-2}	5.41 x 10^{-3}	–	4.76 x 10^{-4}	9.25 x 10^{-2}	2.22 x 10^{-4}	1.324
L	Adult	–	1.90 x 10^{-2}	4.62 x10^{-3}	–	3.87 x 10^{-3}	6.63 x 10^{-2}	6.67 x 10^{-4}	7.71 x 10^{-1}
	Child	–	2.22 x 10^{-2}	5.41 x 10^{-3}		4.6 x 10^{-3}	1.02 x 10^{-1}	7.77 x 10^{-4}	9.0 x 10^{-1}

Table S3. Carcinogenic health risk of heavy metals from groundwater samples in Mankweng.

Site	Individual	Metal			Metal
		**Cd**	**Cr**	**Pb**	
A	Adult	–	1.57 x 10^{-4}	2.43 x 10^{-6}	1.81 x 10^{-4}
	Child	–	1.84 x 10^{-4}	2.83 x10^{-6}	2.12 x 10^{-4}
B	Adult	–	2.86 x 10^{-5}	2.18 x 10^{-6}	3.14 x 10^{-5}
	Child	–	3.34 x 10^{-5}	2.55 x 10^{-6}	3.60 x 10^{-5}
C	Adult	–	1.43 x 10^{-4}	2.43 x 10^{-6}	1.45 x 10^{-4}
	Child	–	1.67 x 10^{-4}	2.83 x 10^{-6}	1.70 x 10^{-4}
D	Adult	–	1.0 x 10^{-4}	2.18 x 10^{-6}	1.02 x 10^{-4}
	Child	–	1.17 x 10^{-4}	2.55 x 10^{-6}	1.20 x 10^{-4}
E	Adult	–	1.86 x 10^{-4}	2.67 x 10^{-6}	1.89 x 10^{-4}
	Child	–	2.17 x 10^{-4}	3.12 x 10^{-6}	2.20 x 10^{-4}
F	Adult	–	2.86 x 10^{-5}	2.43 x 10^{-6}	3.10 x 10^{-5}
	Child	–	3.34 x 10^{-5}	2.83 x 10^{-6}	3.62 x 10^{-5}
G	Adult	–	1.57 x 10^{-4}	2.67 x 10^{-6}	1.60 x 10^{-4}
	Child	–	1.84 x 10^{-4}	3.13 x 10^{-6}	1.87 x 10^{-4}
H	Adult	–	2.86 x 10^{-5}	2.18 x 10^{-6}	3.08 x 10^{-5}
	Child	–	3.34 x 10^{-5}	2.55 x 10^{-6}	3.60 x 10^{-5}
I	Adult	–	5.7 x 10^{-5}	2.43 x 10^{-6}	5.94 x 10^{-5}
	Child	–	6.65 x 10^{-5}	2.83 x 10^{-6}	6.93 x 10^{-5}
J	Adult	2.17 x 10^{-5}	2.86 x 10^{-5}	1.95 x 10^{-6}	5.22 x 10^{-5}
	Child	2.53 x 10^{-5}	3.34 x 10^{-5}	2.09 x 10^{-6}	6.08 x 10^{-5}
K	Adult	–	5,7 x 10^{-5}	2.43 x 10^{-6}	5.94 x 10^{-5}
	Child	–	6.65 x 10^{-5}	2.83 x 10^{-6}	6.93 x 10^{-5}
L	Adult	–	2.86 x 10^{-5}	1.95 x 10^{-6}	3.06 x 10^{-5}
	Child	–	3.34 x 10^{-5}	2.09 x 10^{-6}	3.55 x 10^{-5}
